# Co-localization of acinar markers and insulin in pancreatic cells of subjects with type 2 diabetes

**DOI:** 10.1371/journal.pone.0179398

**Published:** 2017-06-15

**Authors:** Matilde Masini, Lorella Marselli, Eddy Himpe, Luisa Martino, Marco Bugliani, Mara Suleiman, Ugo Boggi, Franco Filipponi, Margherita Occhipinti, Luc Bouwens, Vincenzo De Tata, Piero Marchetti

**Affiliations:** 1 Department of Translational Research and New Technologies in Medicine and Surgery, University of Pisa, Pisa, Italy; 2 Department of Clinical and Experimental Medicine, Islet Cell Laboratory, University of Pisa, Pisa, Italy; 3 Cell Differentiation Laboratory, Vrije Universiteit Brussel (VUB), Brussels, Belgium; Centro Nacional de Investigaciones Oncologicas, SPAIN

## Abstract

To search for clues suggesting that beta cells may generate by transdifferentiation in humans, we assessed the presence of cells double positive for exocrine (amylase, carboxypeptidase A) and endocrine (insulin) markers in the pancreas of non-diabetic individuals (ND) and patients with type 2 diabetes (T2D). Samples from twelve ND and twelve matched T2D multiorgan donors were studied by electron microscopy, including amylase and insulin immunogold labeling; carboxypeptidase A immunofluorescence light microscopy assessment was also performed. In the pancreas from four T2D donors, cells containing both zymogen-like and insulin-like granules were observed, scattered in the exocrine compartment. Nature of granules was confirmed by immunogold labeling for amylase and insulin. Double positive cells ranged from 0.82 to 1.74 per mm^2^, corresponding to 0.26±0.045% of the counted exocrine cells. Intriguingly, cells of the innate immune systems (mast cells and/or macrophages) were adjacent to 33.3±13.6% of these hybrid cells. No cells showing co-localization of amylase and insulin were found in ND samples by electron microscopy. Similarly, cells containing both carboxypeptidase A and insulin were more frequently observed in the diabetic pancreata. These results demonstrate more abundant presence of cells containing both acinar markers and insulin in the pancreas of T2D subjects, which suggests possible conversion from one cellular type to the other and specific association with the diseased condition.

## Introduction

Type 2 diabetes (T2D) is characterized by defects of beta cell mass and function [1]. Although possibly overestimated [2], the loss of beta cell mass is likely due to increased apoptosis and other forms of cell death, associated with inadequate regeneration [1,3]. Nevertheless, some studies have suggested increased beta cell neogenesis (formation of new beta cells from precursors) in human T2D, obesity and pregnancy (reviewed in [3]). One possibility is that beta cells may generate through transdifferentation (i.e. conversion from a different, fully differentiated cell type) from endocrine and non-endocrine pancreatic cells [1,4–6]. Genetic lineage tracing experiments to determine the origin of new beta cells [7–9] can not be performed in the human setting. However, observational studies with the pancreas of non-diabetic and T2D subjects show the presence of endocrine cells containing both glucagon and insulin granules [10–12], which may be interpreted as the conversion of some beta to alpha cells or vice versa, as more clearly demonstrated in rodent models [13,14]. In addition, anecdoctal evidence has been previously reported of the presence of single cells in the pancreas of one healthy subject, containing zymogen granules and a few granules resembling the insulin ones [15]. Similarly, acinar cells with also glucagon granules have been described in two individuals with type 1 diabetes [16]. In agreement with these latter findings, work from a few laboratories has demonstrated *ex vivo* formation of beta cell like cells from human acinar tissue by the use of different experimental approaches [17,18]. However, clues are missing on whether this may be the case *in vivo* in the human T2D condition. In the present study we performed ultrastructural and immunocytochemistry evaluations of pancreatic samples from 12 non-diabetic (ND) and 12 matched T2D multiorgan donors to show the presence of cells containing both acinar cell markers and insulin, that were more frequent in the pancreas of subjects with T2D, suggesting that transition from one cell type to the other could occur *in vivo*.

## Methods

Pancreatic samples from 24 multiorgan donors (12 ND: age, 65.0±3.05 years; BMI, 27.3±0.99 kg/m^2^; and 12 matched T2D: age, 69.0±2.55 years; BMI, 27.2±0.97 kg/m^2^) were obtained with informed written consent and processed according to the procedures previously detailed [19,20], with the approval of the local ethics committee of the Pisa University. Causes of death were similar in both donor groups: trauma, two and three in the ND and T2D group respectively; cardiovascular events, ten and nine. Pancreas cold ischaemia time was <18 h in both groups. In the diabetic group, known duration of the disease was 12.3±2.5 years and all the patients were anti-GAD autoantibody negative. Three patients with diabetes were treated with metformin alone, one with a sulphonylurea alone, five with combined sulphonylurea and metformin, and two with insulin therapy.

Electron microscopy studies were performed as previously described [21,22], with pancreatic samples taken in all the cases at the level of the neck of the gland (before accomplishment of islet isolation, as done routinely in our unit with pancreata not suitable for whole organ transplantation). Non-endocrine and endocrine cells were identified based on their ultrastructural appearance, including the presence of the respective, typical secretory granules [21,22]. Double immunogold labeling for amylase and insulin was performed according to Bendayan [23] by the use of guinea pig anti-insulin and rabbit anti-human α-amylase antibodies (Sigma Aldrich, St. Louis, MO, USA), together with protein A conjugated gold particles (Agar Scientific, Stansted, UK), sized 15 nm for insulin and 5 nm for amylase. For each pancreas, 4 tissue blocks and 33–36 non-consecutive ultra-thin sections were analyzed. Cells of the immune system (macrophages, lymphocytes and mastcells) were classified according to their peculiar electron microscopy characteristics, as previously described by us and others [22,24–26].

Immunofluorescence studies were performed as described [27]. Briefly, pancreatic paraffin sections were immunostained with polyclonal guinea pig anti-insulin (1/3000, gift from prof. C. Van Schravendijk, Brussels), and rabbit anti-carboxypeptidase A (1/1000, AbD Serotec 1810–0006, Oxford, UK). Secondary antibodies were FITC-labeled donkey anti-guinea pig and Alexa Fluor 594-labeled donkey anti-rabbit diluted 1/200 (Jackson Immuno Research, West Grove, USA). Negative controls consisted in omitting the primary antibody. Immunofluorescence was analyzed with a Zeiss LSM710 confocal microscope (Carl Zeiss, Jena, Germany).

Data are expressed as mean±SEM and the two-tailed Student’s t-test was used for statistical comparison of the results.

## Results

**Fig 1:**
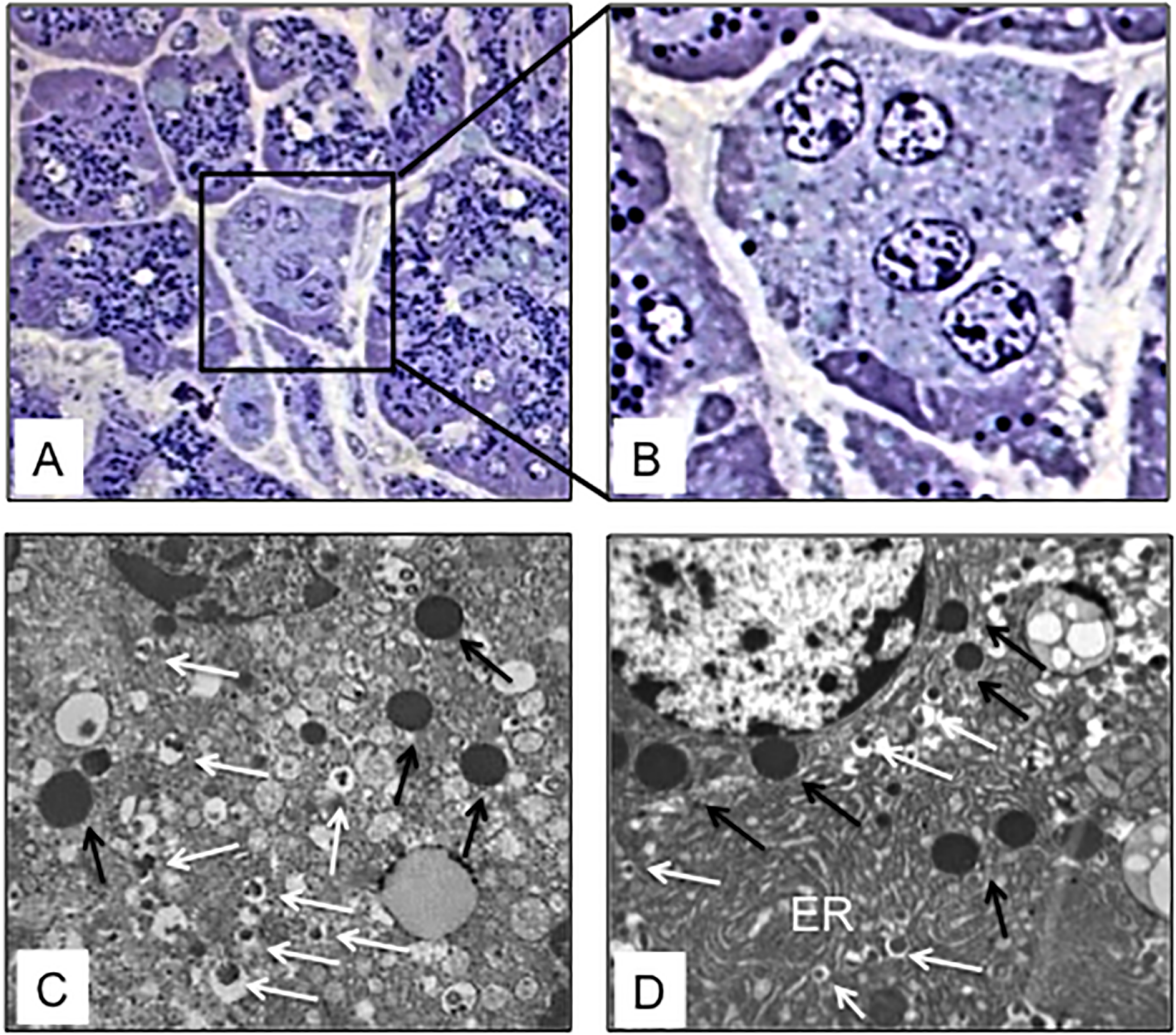
Representative semithin and electron microscopy images of pancreatic cells in T2D slides. A: Semithin section (stained with a 1:1 mixture of toluidine blue, 1% in bidistilled water, and methylen blue, 1% in bidistilled water) showing a cluster of 4 cells with islet-like appearance (magnification: 1,000x), enlarged in B (N: nucleus); C and D: Electron microscopy of cells containing both zymogen-like (black arrows) and insulin-like (white arrows) granules (magnification: 10,000x). ER in D indicates the endoplasmic reticulum, looking expanded and convoluted in this specific T2D beta cell.

**Fig 2:**
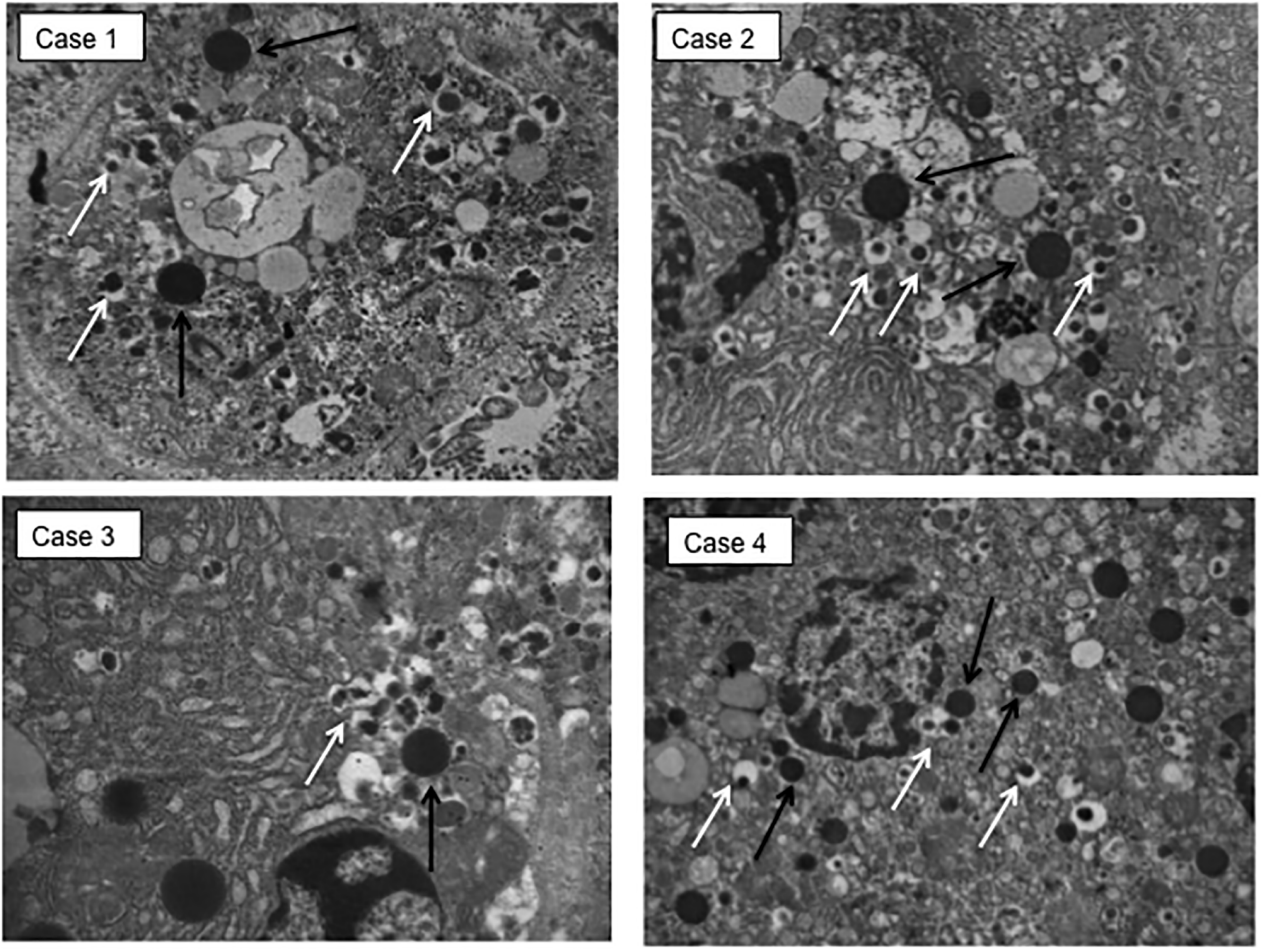
Electron microscopy images (magnification 10,000x) of pancreatic cells containing both zymogen-like (black arrows) and insulin-like (white arrows) granules in the four cases with T2D.

**Fig 3:**
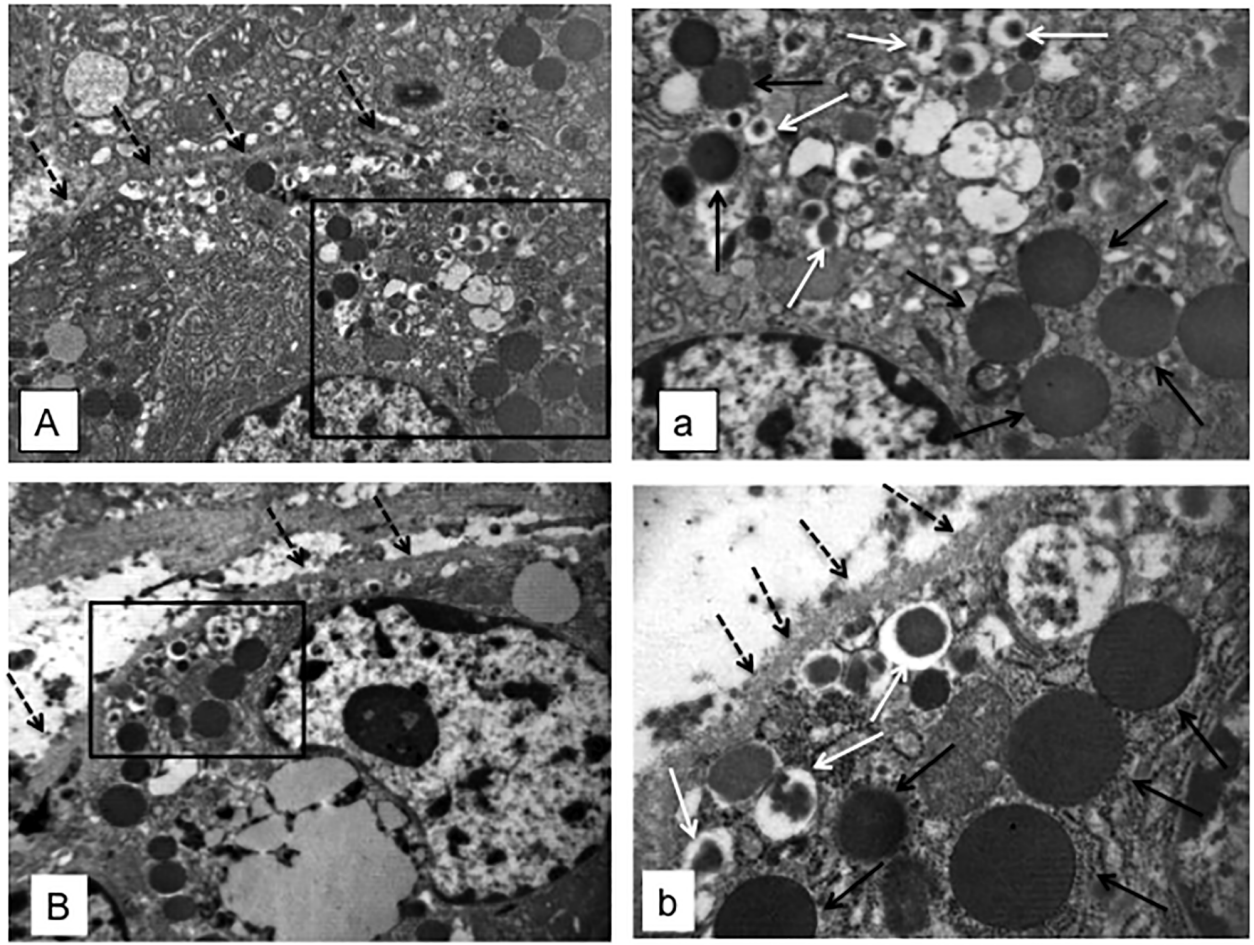
Electron microscopy images (A and B, magnification 10,000x, with squares enlarged in a and b, respectively) showing pancreatic cells in T2D samples, containing both zymogen-like (black arrows) and insulin-like (white arrows) granules, and with cell membrane indicated by the dotted black arrows.

**Fig 4:**
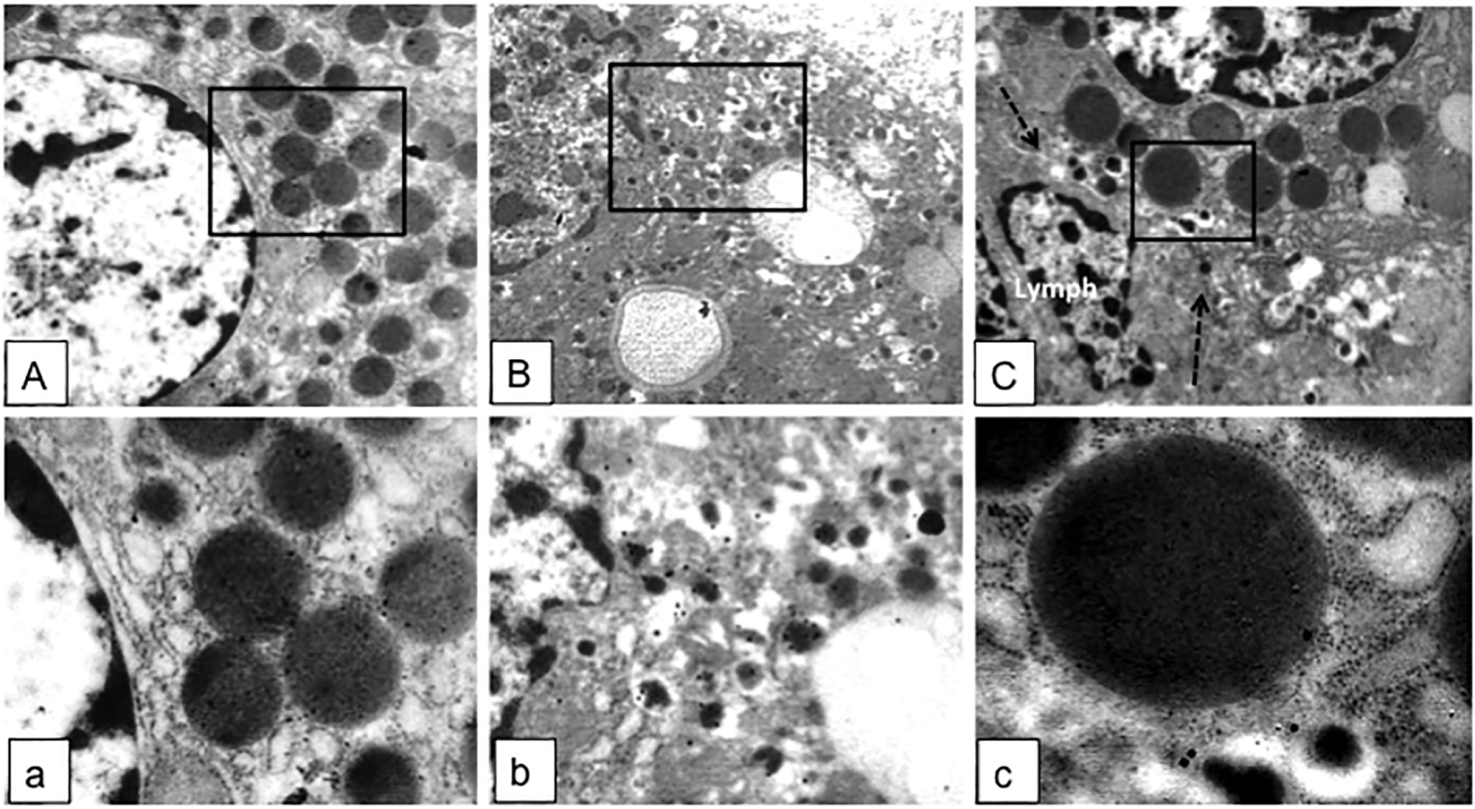
Immunogold labeling of amylase (particle size: 5 nm) and/or insulin (particle size: 15 nm) in an acinar cell (A, square enlarged in a, showing only amylase particles), a beta cell (B, square enlarged in b, showing only insulin particles), and a hybrid cell (C, square enlarged in c, showing both amylase and insulin particles) in a T2D sample. A, B and C: magnification 38,000x; the cell membrane is indicated by the dotted black arrows. Lymph indicates a lymphocyte close to the hybrid cell (see also Fig 7 for additional images of cells of the immune system).

**Fig 5:**
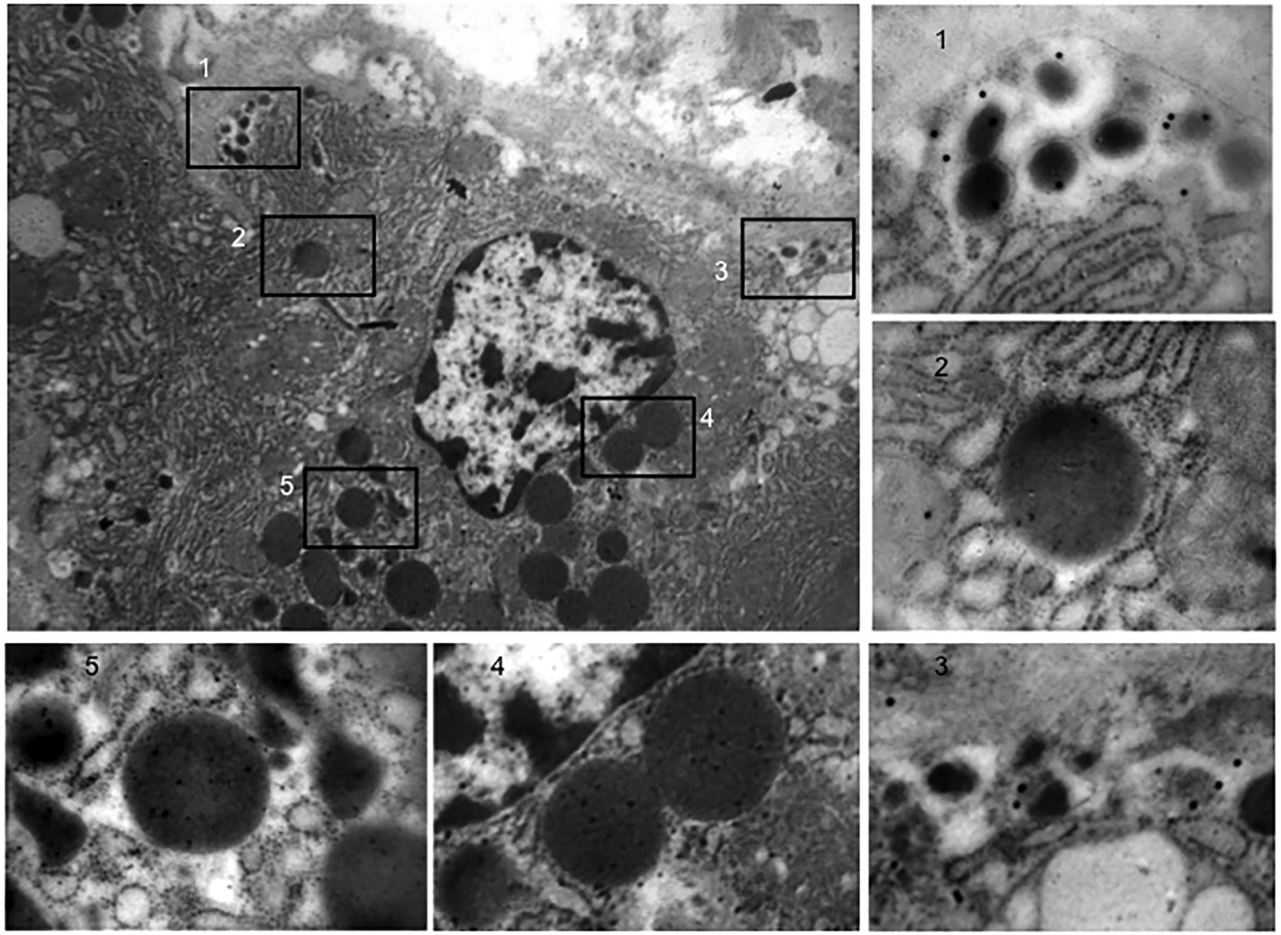
Immunogold labeling of amylase (particle size: 5 nm) and insulin (particle size: 15 nm) of an hybrid cell (magnification: 38,000x) in T2D samples, showing different cellular areas (numbered squares are enlarged) with both positivities in the cytoplasm.

**Fig 6:**
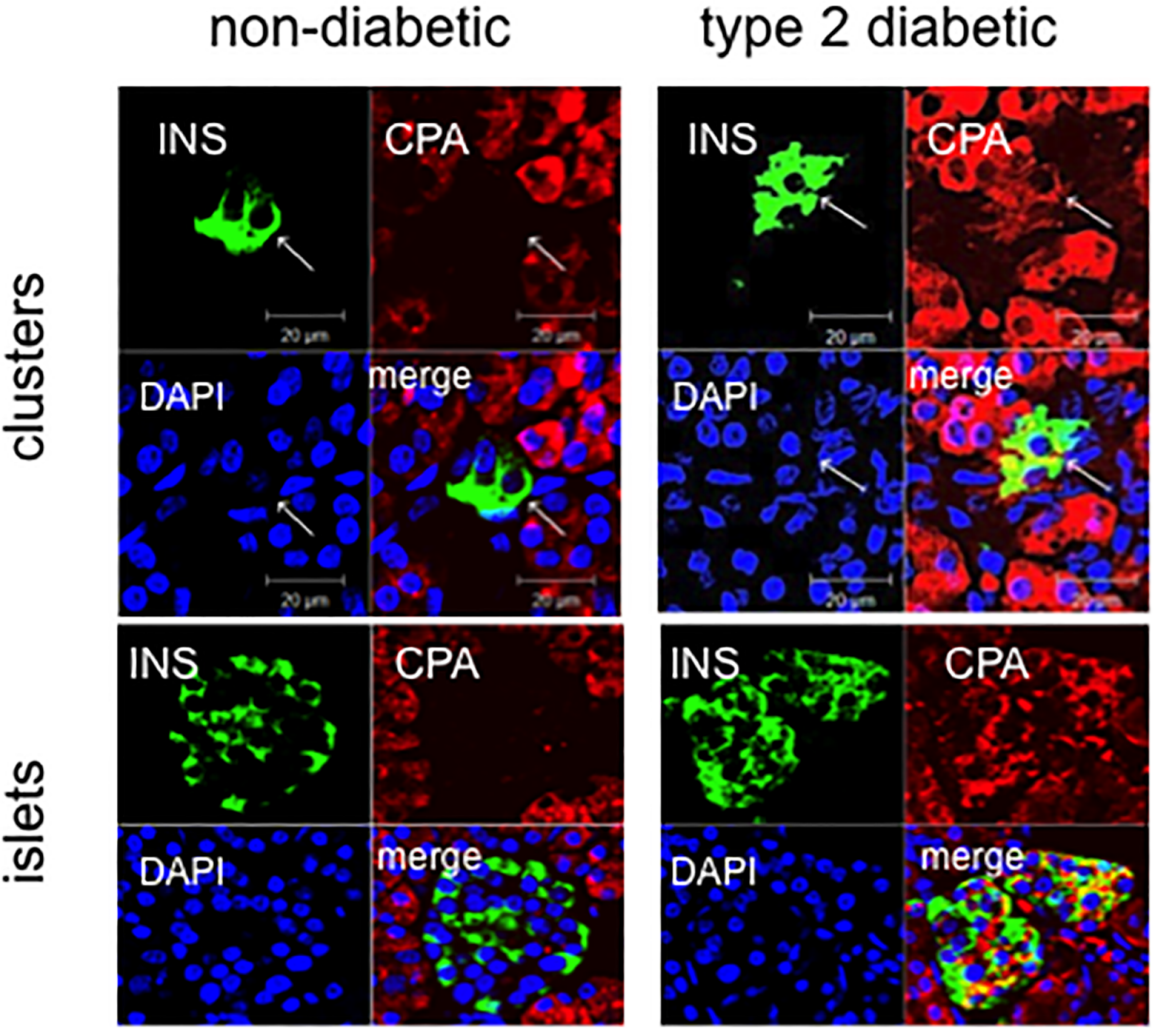
Confocal microscopy images of double immunohistochemical stainings for insulin (green), the acinar enzyme carboxypeptidase A (red) or both the markers (yellow in merge panels). Blue staining is DNA (DAPI, Hoechst dye. Representative cells are shown from a control non-diabetic subject (left panels) and from a subject with type 2 diabetes (right panel).

The pancreatic area that was examined by electron microscopy was 2.67±0.35 and 2.76±0.47 mm^2^ for ND and type 2 diabetes samples, respectively. Semithin sections were first prepared to localize islets and endocrine cell clusters (Fig 1A and 1B), which were then analyzed by electron microscopy (Fig 1C and 1D, Figs 2 and 3). In sections of pancreatic tissue from 4 out of the total 12 T2D donors (but in none of those from ND subjects), and within small clusters of cells with islet-like appearance scattered in the acinar tissue, a few cells were observed, containing both zymogen-like and insulin-like granules in their cytoplasm (Fig 1C and 1D, Figs 2 and 3). By this electron microscopy approach, no cell within the islets (29 islets examined, 5,655 endocrine cells analyzed) was found to contain both zymogen- and insulin-like granules. When the immunogold technique was applied with the simultaneous utilization of anti-amylase and anti-insulin antibodies together with protein A conjugated gold particles of different size (5 and 15 nm, respectively), the zymogen-like granules were labeled for amylase whereas the insulin-like granules were indeed positive for insulin (Figs 4 and 5). Overall, 8,113 (range: 1,123–3,257) acinar cells were counted in these four T2D cases, of which 20 (range: 3–9 per case) contained both types of granules (corresponding to 0.26±0.045% of acinar cells, range: 0.15 to 0.37%). When normalized to the pancreatic areas analyzed, the hybrid cells were 1.34±0.23 per mm^2^ (range: 0.82–1.74). To implement the electron microscopy findings, paraffin sections from the pancreas of 6 ND and 7 T2D donors were also analyzed immunohistochemically by confocal microscopy for the expression of insulin and the exocrine acinar cell marker carboxypeptidase-A. With this approach, cells positive for both carboxypeptidase-A and insulin were commonly found both in endocrine clusters and, more frequently so, within the islets (Fig 6, S1 and S2 Figs). Overall, 27.3±2.7 clusters and islets were examined per pancreas, and cells containing both proteins were 3–4 fold more numerous in the T2D (74.9±2.5% of the analyzed structures) than the ND (22.5±9.3%) samples (p<0.01).

**Fig 7:**
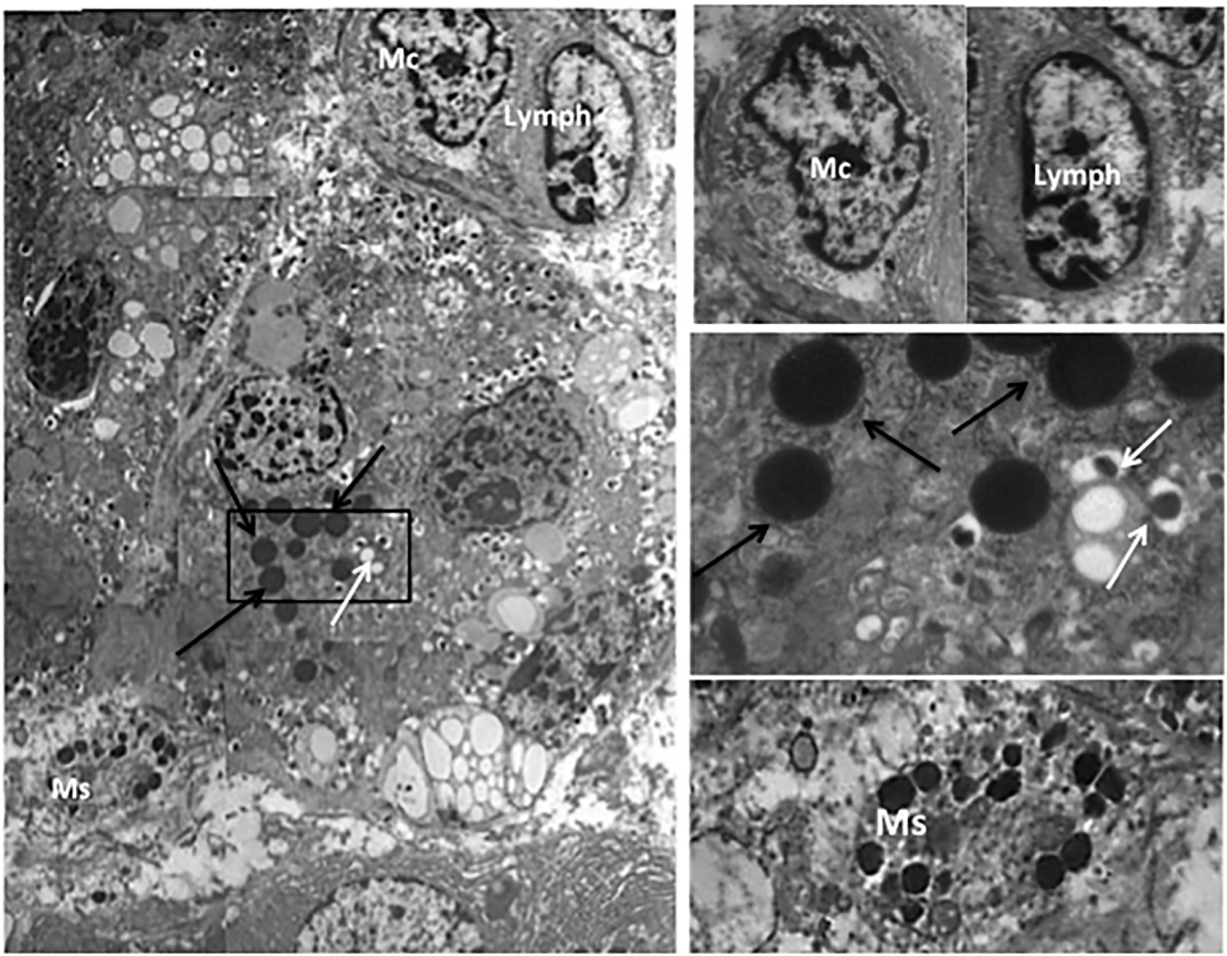
Representative image (left panel) of a pancreatic cell in T2D samples containing both amylase (black arrows) and insulin (white arrows) granules, with adjacent cells of the immune system. The image has been reconstructed from several pictures at 10,000x final magnification. Enlargments are provided in the right panels. Mc: macrophage, Lymph: lymphocyte, Ms: mast cell.

Intriguingly, by electron microscopy cells of the immune system (mainly mast cells and/or macrophages, more rarely lymphocytes) adjacent to the cells containing zymogen and insulin granules were observed in 33.3±13.6% (range: 0–66.6%) of cases. More precisely, mast cells were seen in 19.4±8.4% and macrophage in 13.9±8.3% of cases. Usually, the ratio of hybrid cells to immune system cells was 1:1; however, in one case, 3 cells of the immune system were identified close to 1 mixed cell (Fig 7). Occasional single beta cells (0.23±0.17 per mm^2^) were also observed scattered in the acinar tissue (S3 Fig); however, none of these single beta cells was seen with adjacent cells of immune system.

The subgroup of T2D donors showing pancreatic cells double positive for acinar and endocrine (insulin) markers at the electron microscopy analyses had similar age (70±2.9 vs 67.6±3.27 yrs), BMI (26.7±0.48 vs 27.5±1.2 kg/m^2^) and known duration of diabetes (14.2±5.5 vs 11.1±2.2 yrs) as the subgroup of T2D individuals in whom this type of cells was not observed. In addition, average plasma glucose values during the period of intensive care unit stay were also similar in the two series: 10.4±1.2 vs 11.7±1.0 mmol/L (187±22 vs 210±18 mg/dl). The antidiabetic therapy of the four T2D subjects with double positive cells was metformin alone (1 case), sulphonylurea alone (1 case) or metformin plus sulphonylurea (2 cases).

## Discussion

By performing electron microscopy and immunofluorescence studies, this report shows for the first time that cells double positive for markers of acinar cells and insulin can be found in the human pancreas, with increased abundance in T2D individuals. The presence of cells with both exocrine and endocrine granules as observed by electron microscopy was first described in animal models [28]. Successively, evidence has been reported of single cells in the pancreas of one healthy subject, containing both zymogen and insulin resembling granules [15], and of acinar cells in two individuals with type 1 diabetes with also glucagon granules [16]. Here we report co-localization of zymogen- and insulin granules in pancreatic cells of a subgroup of T2D subjects. These cells were found in small endocrine clusters scattered in the acinar tissue, but not in the islets. Although limited to a small portion of the pancreatic gland, the electron microscopy results of the present study indicate that the quantity of these hybrid cells (appoximately 0.25% of acinar cells) could not be negligible, considering that, in humans, beta cell mass is approximately 1–2% of the total pancreatic mass [1,3,29,30]. It can not be excluded that in other parts of the pancreas (such as the tail portion, where some studies have reported the presence of an increased amount of islets [31]) these figures can be different. In addition, when the presence of carboxypeptidase A was also investigated by immunofluorescence techniques, we found that the amount of cells co-expressing this acinar tissue marker [32,33] and insulin was significantly higher in T2D sample as well. Interestingly, carboxypeptidase A is upregulated in islet beta cells from T2D patients obtained by laser capture microdissection, compared to non-diabetic controls [34], and it is expressed in insulin producing cells derived from embryonic stem cells [35].

Unexpectedly, our observation identified cells of the immune system (mainly mast cells and macrophages) adjacent to the majority of the cells positive for both acinar and insulin granules. Increased immune cell infiltration of pancreatic islets is considered a marker of inflammation, associated with beta cell functional and survival alterations in type 2 diabetes [21,36,37]. However, depending on features of the infiltrating cells and expressed cytokines, “inflammation” may also have beneficial action, including a contribution to pancreas development and cell repair/regeneration [38–40].

Therefore, although indirectly, our findings suggest that under certain circumstances beta cells may derive from pancreatic acinar cells by transdifferentiation, potentially leading to beta cell regeneration in the human pancreas. Transdifferentiation phenomena have been well described and clearly demonstrated in experimental models [5,6,9,14,41–43], and work *ex vivo* with human acinar cells have suggested the feasibility of reprogramming pancreatic non-endocrine cells toward an insulin producing cell phenotype [17,18]. The molecular mechanisms associated with the reprogramming of human acinar cells to insulin containing cells are not fully clear, but previous work in murine models by viral transduction has shown that neurogenin 3, PDX-1 and MafA are likely to play key roles [41,42]. In addition, in their recent *ex vivo* experiments, Lemper et al used lentiviruses expressing activated mitogen-activated protein kinase (MAPK) and signal transducer and activator of transcription 3 (STAT3) to redirect human acinar cells to beta cell like products [17]. Alternatively, Klein et al used exposure to bone morphogenetic protein 7 (BMP7) to induce conversion of non-endocrine cells to insulin producing cells [18]. These experiments were conducted with tissue derived from non-diabetic donors, and it remains to be assessed if these or other mechanisms may be involved in the *in vivo* diabetic human situation.

Intriguingly, we found more cells containing both amylase and insulin, or both carboxypeptidase A and insulin, in T2D than ND pancreatic samples. These differences may reflect biological diversities. For instance, it has been reported that a “neogenic” index calculated on the number of insulin positive cells/clusters was significantly higher in subgroups of type 2 diabetic than in ND organ donors [29]. In addition, our electron microscopy results showed that 1/3 of type 2 diabetic pancreatic samples showed cells with both amylase and insulin. This does not seem to be due to differences in the main clinical features of the T2D donors (age, gender, BMI, known duration of diabetes, blood glucose values during the ICU stay), nor to the type of anti-diabetic therapy at time of death. One possibility is that blood glucose control over a longer period of time (an information not available in our study) may have played a role. For instance, insulin positive area has been reported to correlate negatively with HbA1c levels in donors at the time of death [44].

In conclusion, our results indicate that in subjects with type 2 diabetes more pancreatic cells possess features of both the acinar and beta cell phenotype, in comparison with non-diabetic controls, suggesting previously unseen elements of *in vivo* pancreatic cell plasticity in humans, that may contribute to beta cell neogenesis. However, our observations can not rule out the possibility that, conversely, beta cells may transdifferentiate into acinar cells. Whether our present findings may be applied to other forms of diabetes, including type 1 diabetes, and if the possibility exists that cells positive for acinar markers and insulin could be differently targeted by the immune system, remain currently unknown.

## Supporting information

S1 Fig (TIFF)

S2 Fig (TIFF)

S3 Fig (TIFF)
