# Supplementary figures and images for: Co-localization of acinar markers and insulin in pancreatic cells of subjects with type 2 diabetes
Source: PLoS One. 2017 Jun 15;12(6):e0179398. doi: 10.1371/journal.pone.0179398 (PMC5472296; doi:10.1371/journal.pone.0179398)

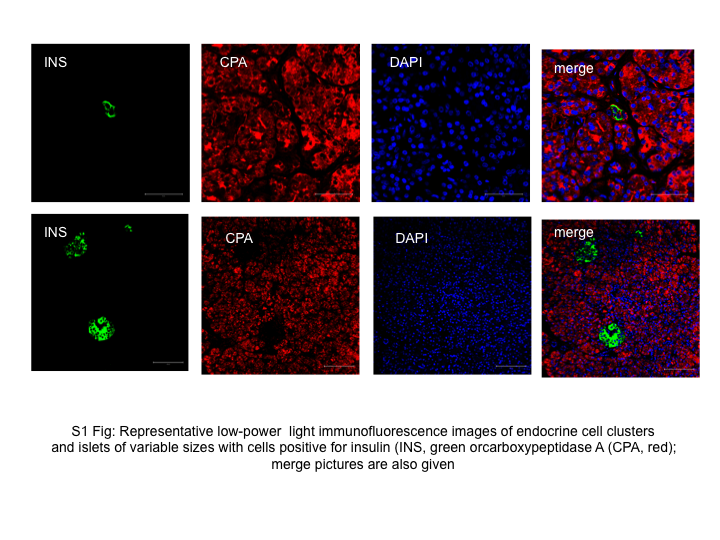

Supplement: S1 Fig — (TIFF) [file pone.0179398.s001.tiff]

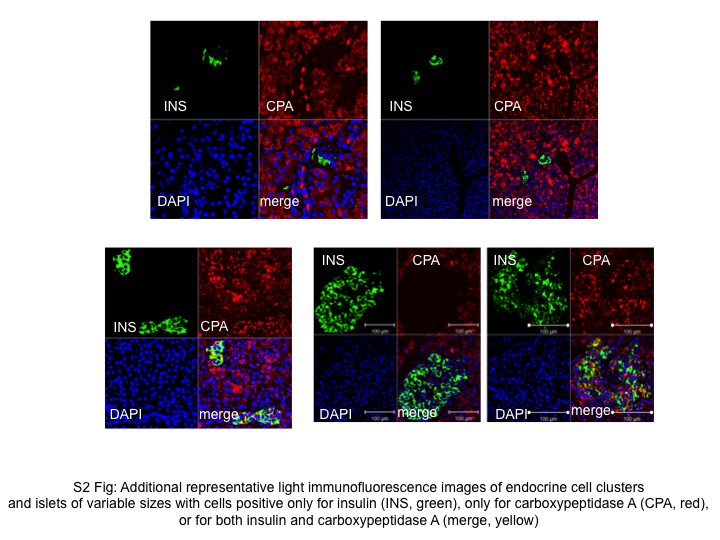

Supplement: S2 Fig — (TIFF) [file pone.0179398.s002.tiff]
